# Resolvin D2 induces anti-microbial mechanisms in a model of infectious peritonitis and secondary lung infection

**DOI:** 10.3389/fimmu.2022.1011944

**Published:** 2022-12-01

**Authors:** Prem Y. Kadiyam Sundarasivarao, Jean M. Walker, Ana Rodriguez, Bernd W. Spur, Kingsley Yin

**Affiliations:** Department of Cell Biology and Neuroscience, Rowan University School of Osteopathic Medicine, Stratford, NJ, United States

**Keywords:** inflammation, neutrophils, myeloid-derived suppressor cells, *Pseudomonas aeruginosa*, cytokines, specialized pro-resolving mediators, alveolar macrophages, innate immunity

## Abstract

In severe bacterial infections, there is a pro-inflammatory response to promote bacterial clearance but this response can cause tissue injury. Later, the immune system becomes dysregulated and the host is unable to clear a secondary or a pre-existing infection. Specialized Pro-resolving Mediators (SPMs) such as resolvin D2 (RvD2) have been shown to be beneficial for inflammation/infection resolution in animal models of sepsis but *in vivo* mechanisms by which RvD2 may promote bacterial clearance and/or attenuate deleterious effects of a secondary infection have not been fully established. In this study, we used the 2-hit model of cecal ligation and puncture (CLP) induced infectious peritonitis and secondary lung infection with *Pseudomonas aeruginosa* to find possible antimicrobial and immunomodulatory mechanisms of RvD2. We show that RvD2 given as late as 48h after CLP surgery reduced blood bacterial load without altering plasma cytokines compared to mice given saline vehicle. RvD2 increased splenic neutrophil accumulation as well as average reactive oxygen species (ROS) production. There was also an increase in an immature leukocyte population the myeloid derived suppressor cells (MDSCs) in the spleen of RvD2 treated mice. RvD2 reduced lung lavage bacterial load 24h after *P. aeruginosa* administration and significantly decreased lung lavage levels of IL-23, a cytokine essential in the Th-17 inflammatory response. In addition, we show that RvD2 increased the number of non-inflammatory alveolar macrophages after *P. aeruginosa* administration compared to saline treated mice. The study uncovered an antimicrobial mechanism of RvD2 where RvD2 increases mature neutrophil and MDSC accumulation into the spleen to promote blood bacterial clearance. The study showed that in this 2-hit model, RvD2 promotes lung bacterial clearance, increased non-inflammatory alveolar macrophage number and inhibits an adaptive immune pathway providing evidence of its resolution mechanism in secondary pulmonary infection.

## Introduction

Sepsis is an inflammatory disorder caused by an abnormal immune response to infection. An initial hyperinflammatory response to primary infection, can damage tissues leading to multiple organ dysfunction (1). There can follow a dysregulated inflammatory response which plays a central role in development of immunosuppression during sepsis (2, 3). Unlike auto-immune disorders, inflammation in sepsis is triggered by bacterial, viral, or fungal infections. In sepsis, prolonged immunosuppression makes the host susceptible to secondary infections caused by bacteria such as *Pseudomonas aeruginosa (P. aeruginosa)*, *Staphylococcus aureus, Streptococcus pneumoniae, or Escherichia coli* (2–5). Improving host defense during this immunosuppressive phase can prevent secondary infections and ameliorate damage due to hyperinflammation as a result of these infections.

During infection, interaction between many cell types results in the synthesis and secretion of bioactive lipid mediators called Specialized Pro-resolving Mediators (SPMs). SPMs are derived from polyunsaturated fatty acids and include resolvins, lipoxins, maresins, and protectins (6). Lipoxins are synthesized from arachidonic acid (AA) and E-series and D-series resolvins are synthesized from Eicosapentaenoic acid (EPA) and Docosahexaenoic acid (DHA) respectively. Resolvin D2 (RvD2) is a D-series resolvin which binds to G-protein coupled receptors (GPR18) expressed on immune cells of the innate and adaptive immune system (7, 8). RvD2 has been shown to increase survival, reduce systemic inflammation and decrease bacterial load in murine cecal ligation and puncture (CLP) sepsis (9, 10). At the cellular level, RvD2 reduced monocyte/macrophage inflammatory responses, neutrophil migration, and increased macrophage phagocytic ability (11–15). Importantly, analysis of leukocytes from septic patients shows that there is a dysregulation of resolvins D1 and D2 signaling where neutrophils and certain populations of monocytes have reduced phagocytic ability even though there is increased RvD1 and RvD2 receptors (16). The extent of this uncoupling is significantly correlated to clinical severity. This uncoupling was partially reversed with RvD1 and RvD2.

*P. aeruginosa* is an opportunistic gram-negative bacteria commonly found in sepsis patients. We have previously established a 2-hit model of cecal ligation and puncture (CLP) - induced sepsis followed by a secondary lung infection of *P. aeruginosa* (17). Using this model, we investigated the role of RvD2 on host defense mechanisms by administering it during the immunosuppressive phase and further challenged mice lungs with *P. aeruginosa* infection to study if RvD2 administration enabled mice to defend against the ensuing pneumonia. In this model, RvD2 given 48h after initial surgery reduced blood bacteria load without altering blood or tissue cytokines. RvD2 reduced subsequent lung *Pseudomonas* load and inflammation. The mechanisms for these results were not fully elucidated.

The spleen is a secondary lymphoid organ whose function has been implicated in poor sepsis outcomes in both animal models of sepsis as well as in humans. There is evidence that splenectomy patients are more susceptible to sepsis (18, 19). Apoptosis of splenocytes and an increase in T-reg cells have been reported to contribute significantly to immunosuppression in CLP- sepsis (20, 21). Splenic myeloid-derived suppressor cells (MDSCs) have been reported to be increased in the spleen of CLP mice (22, 23). MDSCs are immature, heterogenous populations of granulocytic and monocytic cells (24). These cells have been shown to have immunosuppressive properties where they can suppress T-cell function (25, 26). On the other hand, MDSCs have also been shown to attenuate inflammatory responses and possibly be beneficial in sepsis (22, 27, 28). Furthermore, there is evidence that mature and immature splenic neutrophils contribute significantly to the eradication of bacteria (29).

Secondary lung infections causing pneumonia significantly increase mortality in sepsis patients (30). However cellular changes inside lung during this is poorly understood. Alveolar macrophages that reside in alveolar space in lungs can either contribute to development of inflammation (31) or prevent excessive inflammation (32) in response to influenza viral infection in mice (33). Non-inflammatory alveolar macrophages do not express CD11b (34) but express SiglecF (35). As CD11b expression is involved in leukocyte migration and inflammation, the appearance of non-inflammatory CD11b^-^SiglecF^+^ alveolar macrophages is a marker of infection/inflammation resolution (34). Therefore to understand infection resolution we studied non-inflammatory alveolar macrophages (CD11b^-^ SiglecF^+^).

In the current study, we investigated if RvD2 given 48h after CLP-surgery can alter the number and functionality of splenic cells in a CLP model of mild sepsis with *P. aeruginosa* secondary infection. The study provides evidence that RvD2 increased the number of splenic neutrophils and MDSCs. We show an increased efficiency of reactive oxygen species (ROS) release by splenic neutrophils. These changes were associated with increased blood bacteria clearance without any alterations in plasma cytokine levels. We also show that non-inflammatory alveolar macrophage (CD11b^-^SiglecF^+^) numbers are elevated, and lung lavage inflammatory cytokine release is attenuated in RvD2 treated CLP mice. Together these results provide evidence of splenic neutrophil, MDSC and alveolar macrophage mediated antimicrobial mechanisms for RvD2 primary and secondary infection resolution.

## Methods

All experiments using animals were carried out in complete compliance with protocols approved by the Institutional Animal Care and Use Committee (IACUC) at Rowan University School of Osteopathic Medicine.

### Model of peritonitis

CLP surgery was performed on male C57BL/6 mice (11-13 weeks old; weight range of 24 – 32g) using modified methods as previously published (17). We used male mice because there have been studies which show that female mice had an advantage in survival and different immunological response after CLP (36). On the day of surgery, mice were anesthetized with isoflurane (+ O_2_). A 1 cm-long midline incision was made in the abdomen to expose the cecum. The distal third of the cecum was ligated with 4.0 surgical silk. Using a 27-gauge needle, the cecum was punctured twice, through and through. A small drop of feces was extruded through the holes to ensure patency of the punctures. The cecum was placed back into the abdomen, which was then closed in 2 layers. For sham controls, ceca were removed but not ligated, nor punctured. Saline (8 mL/100 g; s.c.) was injected to replace any fluid loss during surgery. Buprenorphine-SR (1 mg/kg, s.c.) was injected. Mice were monitored every 12 hours. 48 h after surgery, CLP mice were anesthetized and injected with either RvD2 (100 ng/mouse) or saline vehicle *via* the tail vein. 24h later, mice were anesthetized with ketamine/xylazine (100/10 mg/kg, i.p.). Blood was obtained by intracardiac puncture into 1 ml syringes containing 0.05 M EDTA. The blood was centrifuged at 500 X g for 12 min. Plasma was stored at -75°C until analysis. Spleens were taken, cells were isolated and then analyzed by flow cytometry.

### Model of peritonitis *+ Pseudomonas aeruginosa* secondary lung infection

**Figure 1:**
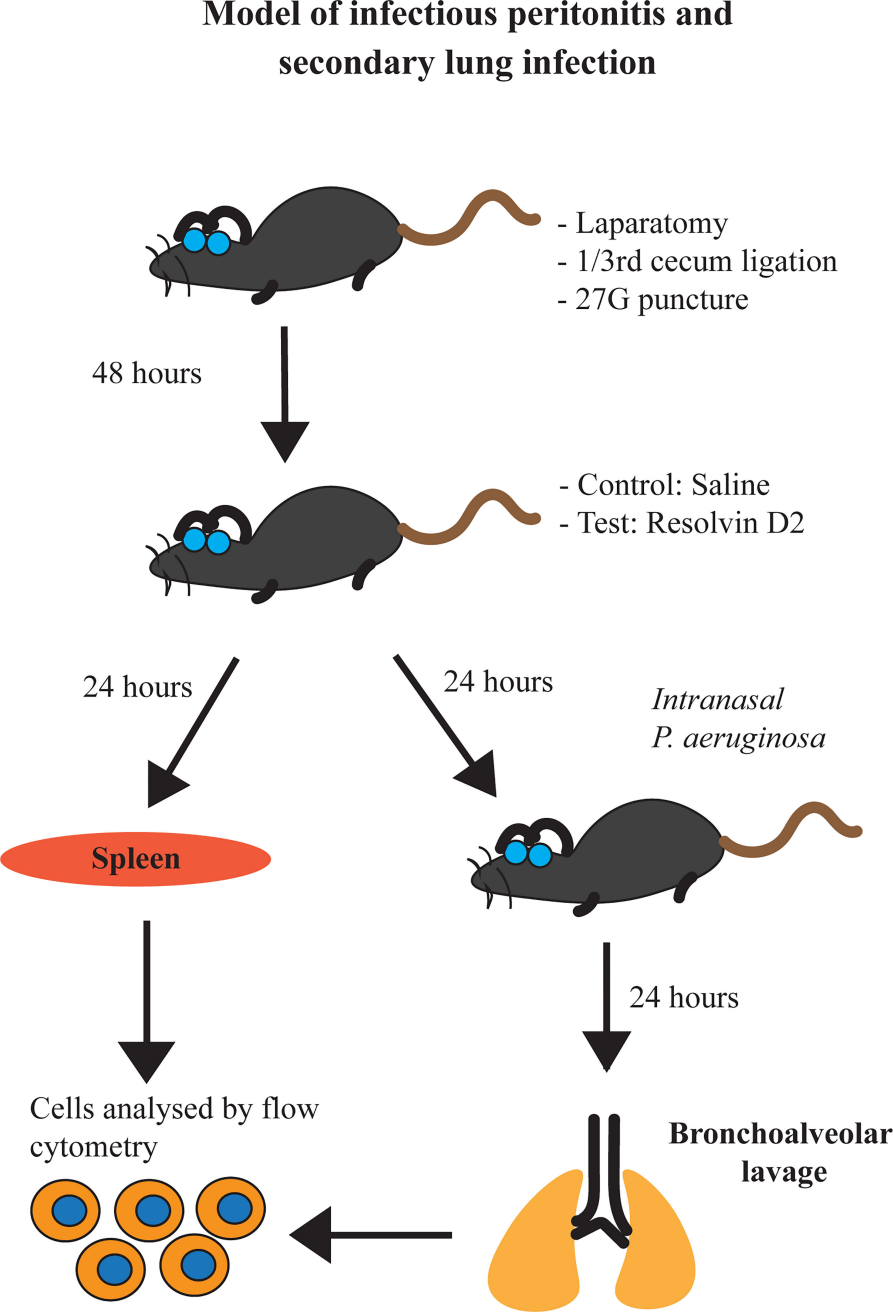
Schematic representation of our model of infectious peritonitis and secondary lung infection.

For this model, mice were anesthetized with isoflurane and *P. aeruginosa* (approx. 10^7^ cells) (ATCC 27853) or saline vehicle was inoculated intranasally. Mice continued to be monitored every 8h. At 24h, post inoculation, mice were sacrificed. Tracheas were cannulated using 23G tubing adaptors. Lungs were then lavaged with 1 ml PBS containing sodium citrate (0.38% final concentration). A flow diagram schematic of our studies is provided (**Figure 1**).

### *Pseudomonas aeruginosa* culture and counting

*Pseudomonas aeruginosa* from the frozen stock (ATCC 27853) was streaked onto a tryptic soy agar (TSA) plate in a Bio-safety level 2 (BSL-2) cabinet and plates are incubated at 37°C for 16-24 hours. After incubation, a few colonies were transferred into sterile tubes containing 3 mL of LB broth media and incubated at 37°C for 3 hours in a shaker rotating at 180rpm. Number of bacterial cells are counted by OD600 reading and CFU plating. 25 μL of 0.2 Abs (OD 600) diluted culture was found to have approximately 10^7^ bacterial cells.

### Bacterial load

1:10 serial dilutions of blood or lung lavage fluid were made and aseptically spread onto tryptic soy agar (TSA) plates to measure aerobic bacteria growth (Sigma-Aldrich, St. Louis, MO). The plates were then incubated overnight at 37°C. The number of bacterial colonies (CFU) was assessed 24 h later by observers blinded to the treatment groups.

### Isolation of cells from spleen

Whole spleens were passed through a 70 μm cell strainer placed inside a petri plate containing 10 ml ice-cold phosphate-buffered saline (PBS). Cells were centrifuged at 600 X g for 8 min at 4°C and supernatant discarded. Cells were then resuspended in 5 ml of ACK cell lysis buffer (Quality Biological, Gaithersburg, MD) and incubated on ice for 5 min to slowdown lysis After incubation, cells were centrifuged at 600 X g for 8 min at 4°C. Cell pellets were resuspended in 2 ml of cell staining buffer (Biolegend, San Diego, CA). Total number of cells was measured using a Countess cell counter (Countess II FL; Invitrogen, Waltham, MA) by mixing 5 µl of cell suspension with 5 µl of 0.4% Trypan blue solution (Corning) and loading 10 µl onto a cell counting chamber slide (Thermo Fisher Scientific, Waltham MA). Gate sizes were adjusted to count cells between 7 µm and 35 µm and to avoid counting cell aggregates. Viability of cells after red blood cell lysis was approximately 80% to 90%.

### Flow cytometry cell staining and analyses

Approximately 1 million cells were aliquoted in separate tubes and Fc receptors were blocked using 1 µl of TruStain FcX (anti-mouse 16/32) antibody (BioLegend, San Diego, CA) for 1 h on ice. Then cells were washed with cell staining buffer and 1 µl of different antibodies including anti-Ly6C, anti-Ly6G, anti-CD11b (all BioLegend, San Diego, CA), and anti-SiglecF (BD Pharmingen, Franklin Lakes, NJ) were added to tubes along with 1 µl of live/dead cell stain (Invitrogen, Waltham, MA) and incubated for 30 min on ice. After incubation, cells were washed with cell staining buffer and resuspended. For flow cytometry compensation of multiple fluorophores, ABC anti-rat capture beads (Invitrogen, Waltham, MA) and ArC amine reactive capture beads (Invitrogen, Waltham, MA) were used for accurate separation of overlapping emission wavelengths of multiple fluorophores used in identification and characterization of cells in spleen and lungs. Approximately, 100,000 – 250,000 cells were analyzed by flow cytometry (Attune acoustic focusing cytometer; Applied Biosystems, Waltham, MA). Compensation beads were used to adjust PMT voltages and compensation was set for multiple fluorophores. Cells were gated based on size (forward scatter) and granularity (side scatter), single population, live cells, and expression of Ly6C, Ly6G, CD11b, and SiglecF proteins (**Supplemental Figure 1** and **Table 1**). FlowJo software (version 10) was used to analyze flow cytometry data.

**Table 1:** Antibodies and stains used for flow cytometry.

Antibody/Stain	Flurophore and Clone	Dilution	Supplier and Cat. #
Ly6C	AF-488; HK 1.4	1:500	Biolegend 128022
Ly6G	AF-647; 1A8	1:500	Biolegend 127610
CD11b	PE; M1/70	1:1000	Biolegend 101207
Siglec-F	PerCP-Cy 5.5; E50-2440	1:500	BD Pharmingen 565526
Live/Dead Cell Stain	Near IR	1:500	Invitrogen L34976

### Oxidative burst assay

Splenic cells were isolated as described previously and approximately 1 million cells were blocked with 1 µl of TruStain FcX (anti-mouse 16/32) antibody (Biolegend, San Diego, CA) for 1 h on ice. After blocking, 1 µl of different antibodies including anti-CD11b, anti-Ly6G, and anti-Gr1 (All BioLegend, San Diego, CA) were added to each tube together with 1 µl of live/dead cell stain (Invitrogen, Waltham, MA). Hanks’ Balanced Salt solution was used for immunostaining, washing, and resuspending cells. Then 4 µl of dihydrorhodamine 123 (DHR; 5 mM) and 10 µl of N-formyl-Met-Leu-Phe (fMLP) bacterial peptide (10 mM) were added to cells, resuspended in 500 µl of Hanks’ Balanced Salt Solution (HBSS), incubated at 37°C for 20 min and then immediately transferred to ice for 10 min to stop the reaction (37). Cells were washed twice with HBSS, and fluorescence intensity was measured by flow cytometry.

### Measurement of blood and lung lavage cytokines using flow cytometry

LEGENDplex mouse inflammation panel kit (BioLegend, San Diego, CA) was used to measure concentrations of different cytokines in blood, This panel included cytokines, interleukin-1β (IL-1β), interleukin-6 (IL-6), interleukin-10 (IL-10), interleukin-17 (IL-17), interleukin-23 (IL-23), monocyte chemoattractant protein-1 (MCP-1), interferon-β (IFN-β), interferon-γ (IFN-γ) and tumor necrosis factor-α (TNF-α). Manufacturer’s recommended protocol was used to incubate capture beads with blood plasma samples or lung lavage fluid of different groups of mice. Around 5000 capture beads were analyzed and bead MFI corresponding to bound analyte was measured by flow cytometry. Then using LEGENDplex software individual fluorescence intensity values were obtained. Unknown sample concentrations were obtained by cubic spline-based interpolation from a standard curve generated from known standards.

### RvD2 synthesis

RvD2 was totally synthesized by previously published methods (38). The purity of the compound was measured by HPLC-Mass Spectrometry and determined to be >98% purity. 10 μg of compounds was dissolved in 400 μl sterile saline on days of experiments.

### Statistical analyses

Student’s t-test was used for comparison of differences in data with 2 groups except for bacteria load. Data for all other measurements were analyzed using one way analysis of variance. Differences between the groups were then determined using Dunnett’s multiple comparisons test of significance. A Mann-Whitney U test was used for comparison of bacterial load. For all analyses, P< 0.05 was taken as significant. All data are expressed as mean ± S.E.M.

## Results

### Tissue analyses after 1^st^ hit

In initial studies, we investigated the effects of late (48h) administration of RvD2 on bacteremia and systemic inflammation after CLP-induced infectious peritonitis. As splenic leukocytes have been shown to be involved in bacterial clearance (29), we also examined the effects of RvD2 on splenic leukocytes. 48 h after CLP surgery, mice were given either RvD2 or saline vehicle. 24 h after these injections, the mice were sacrificed. Blood and spleens were taken for analyses.

### Blood bacterial load

**Figure 2:**
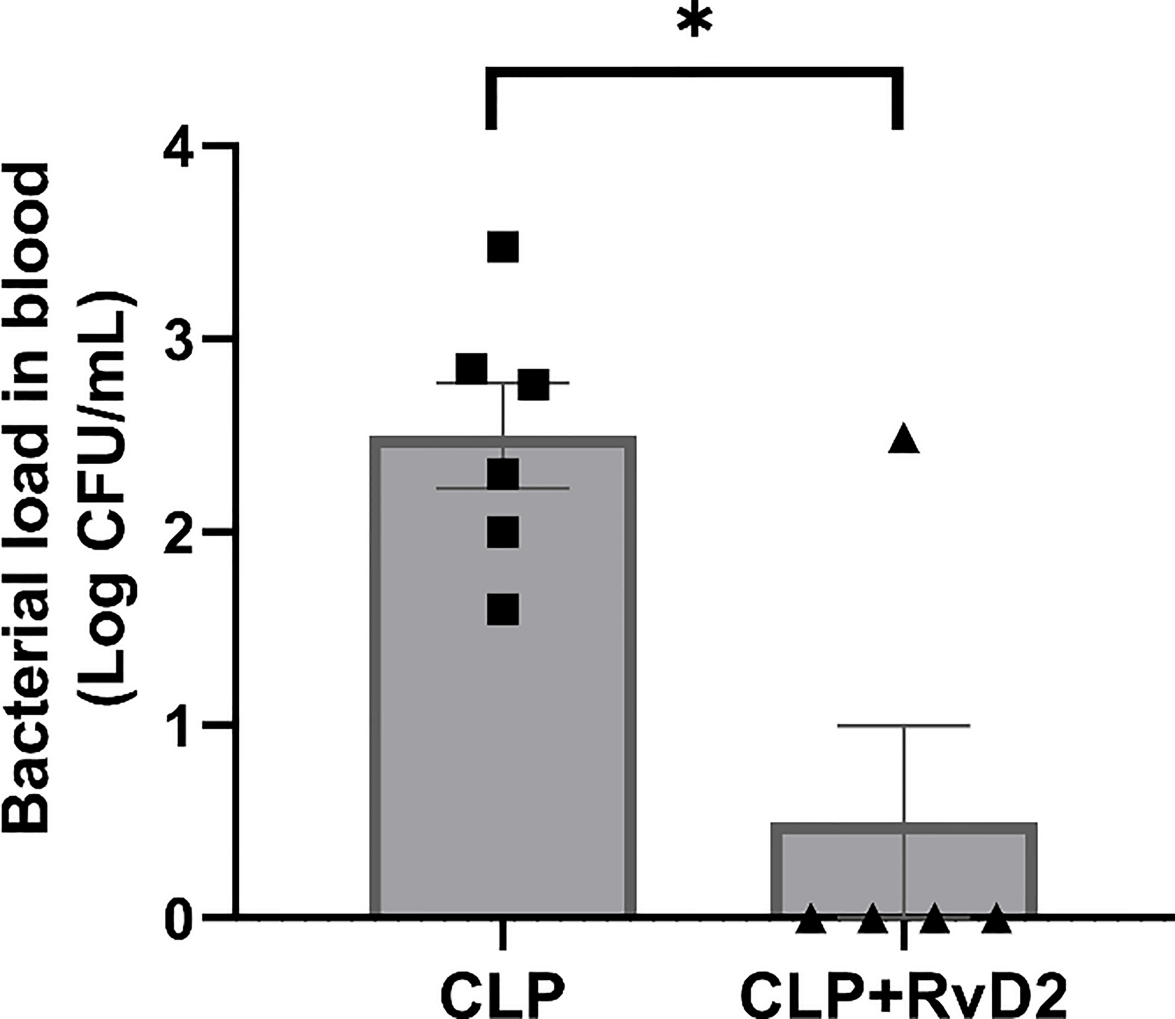
CLP mice were given vehicle saline or RvD2 48h after surgery. Mice were sacrificed 24h later and blood sample were taken. Samples were serially diluted, plated on TSA plates and colony forming units (CFUs) were counted 24 hours after plating. RvD2 reduced the blood bacteria load. * P < 0.05, for n = 5 - 6 mice in each group.

Blood was serially diluted and plated on tryptic soy agar plates. RvD2 treatment of CLP mice significantly reduced blood bacteria load compared to CLP mice given vehicle saline (**Figure 2**).

### Plasma cytokines

**Figure 3:**
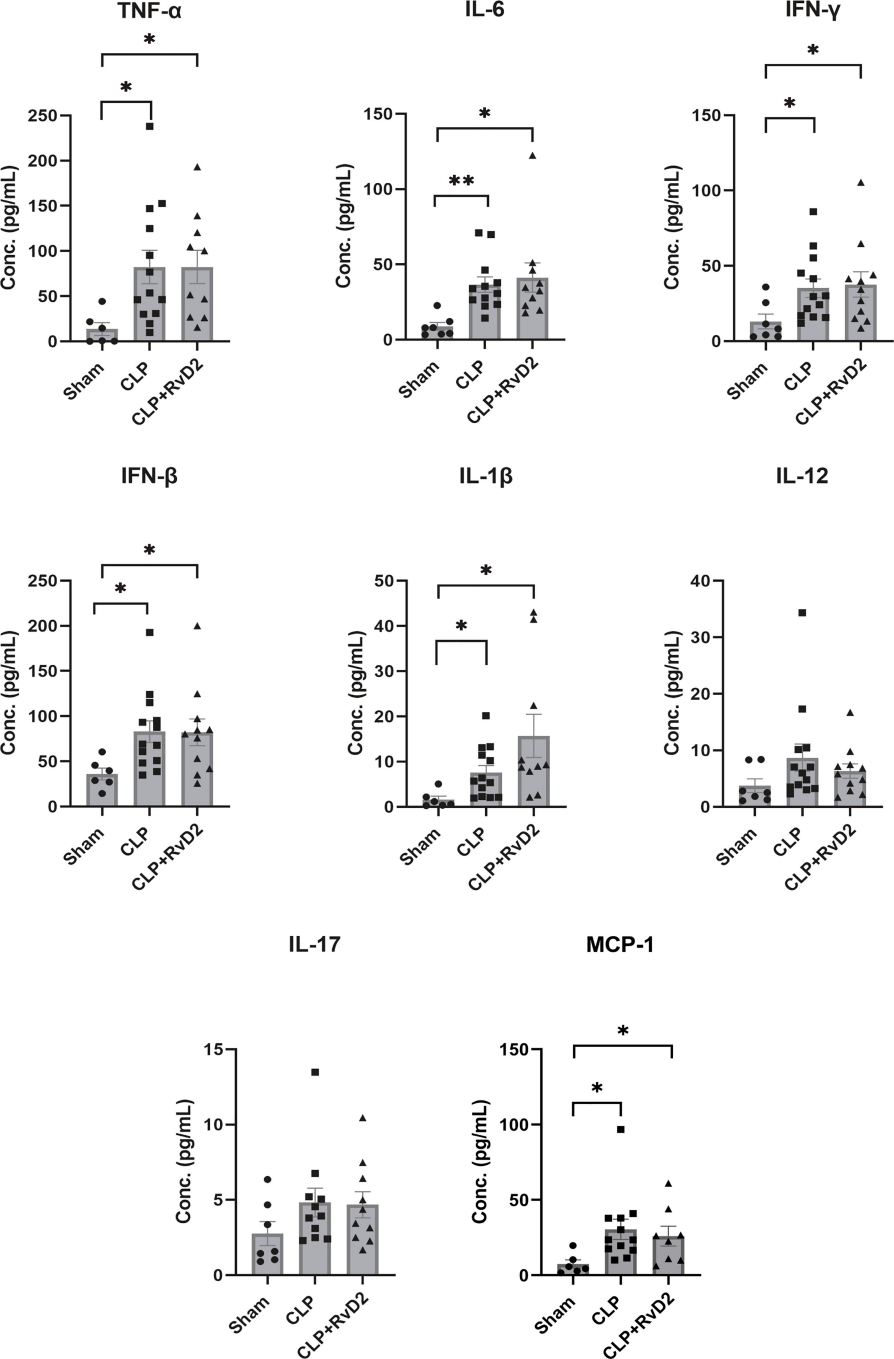
Sham or CLP surgery was performed on mice. CLP mice were given vehicle saline or RvD2 48h after surgery. Mice were sacrificed 24h later and blood samples were taken. Using flow cytometry, cytokines including TNF-α, IL-6, IFN-γ, IFN-β, IL-1β, IL-12, IL-17, and MCP-1 were quantified using bead-based immunoassay. CLP increased plasma levels of TNFα, IL-6. IFN-γ, IFN-β, IL-1β and MCP-1 but RvD2 did not change levels further. * P < 0.05, ** P < 0.01 for n = 6 – 14 in all groups.

Plasma cytokine levels of plasma obtained from mice after the 1^st^ hit (CLP) were measured using the Legendplex system (BioLegend). Plasma levels of TNFα, IL-6, IFN-γ, IFN-β, IL-1β and MCP-1 were increased in CLP mice compared to sham controls, but RvD2 did not significantly affect this increase (**Figure 3**). Plasma levels of IL-17 and IL-12 were not increased in CLP mice compared to sham controls. Elevation of IFN-β, an immunosuppressive cytokine without any elevation in IL-17 and IL-12 both pro-inflammatory cytokines, and all associated with the adaptive immune system, suggest that mice were not in a pro-inflammatory phase (39). This is supported by preliminary work in this model which shows that in the liver, IL-6 (a predominantly pro-inflammatory cytokine) was not significantly elevated over sham controls but IL-10 (an anti-inflammatory cytokine) was increased which suggests that the mice were in a post-pro-inflammatory phase. It is important also to note that elevations above sham in liver cytokine levels (**Supplemental Data**; **Figure 2**) and plasma cytokine levels provide evidence of peritonitis.

### Splenic neutrophils

**Figure 4:**
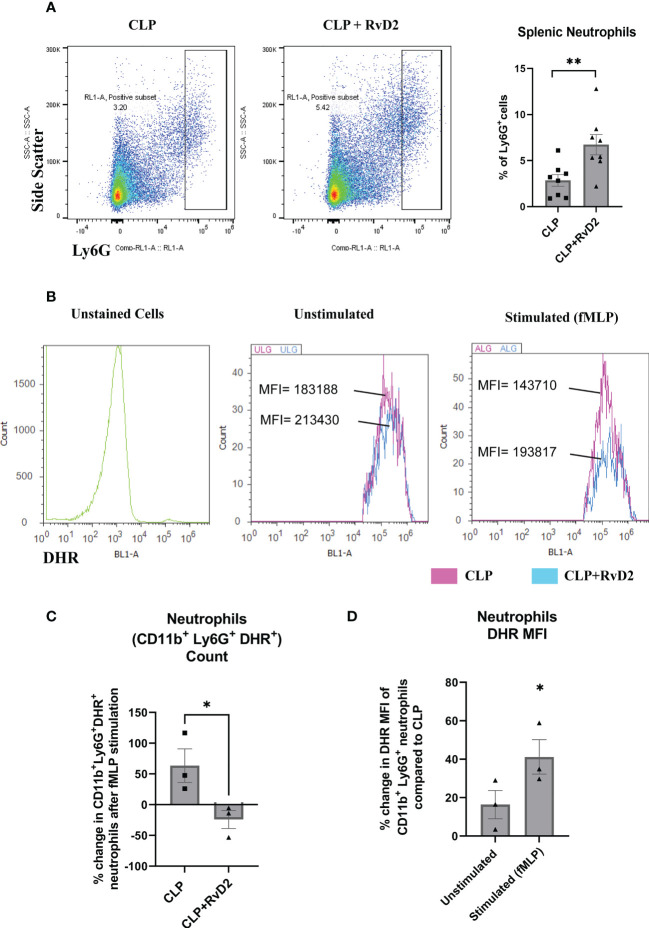
**(A)** CLP mice were given vehicle saline or RvD2 48h after surgery. Mice were sacrificed 24h later and spleen samples were taken. Ly6G^+^ splenic neutrophils were gated and counted based on side scatter and Ly6G expression. RvD2 increased the number of Ly6G^+^ splenic neutrophils. Data are mean ± S.E.M. for n = 8 in each group, **P<0.01. **(B)** ROS production (DHR^+^ cells) in CD11b^+^ Ly6G^+^ splenic neutrophils was measured in unstimulated (basal) and fMLP stimulated cells. Cell density plots (Y-axis: count; X-axis: DHR; oxidative burst) shows oxidative burst in cells taken from CLP and CLP + RvD2 mice. **(C)** The percentage change in the number of the fMLP stimulated neutrophils (CD11b^+^ Ly6G^+^ DHR^+^) was measured. RvD2 decreased the number of fMLP stimulated neutrophils. **(D)** The percentage change in median fluorescence intensity (MFI) in CD11b^+^ Ly6G^+^ DHR^+^ cells (basal and stimulated) between cells taken from CLP+RvD2 treated mice and cells from CLP mice was measured. RvD2 increased the MFI of ROS producing splenic neutrophils. All data are mean ± S.E.M. *P<0.05 for n = 3 in all groups.

Splenic neutrophil numbers were measured using flow cytometry by staining cells with Ly6G. After gating cells by forward and side scatter, singlets, and live cells, gates were set to quantify the percentage of Ly6G^+^ mature splenic neutrophils. **Figure 4A** shows that RvD2 increased percentage of splenic neutrophils. In addition to measuring the number of splenic neutrophils, we also assayed the respiratory burst of splenic neutrophils which are subpopulations of Ly6G^+^ neutrophils that express inflammatory CD11b^+^ marker. The purpose of measuring the oxidative burst is because intracellular reactive oxygen species production is an important part of the bacteria killing process (40). This assay was performed by incubating these splenic cells with dihydrorhodamine which detects intracellular ROS, and bacterial peptide fMLP. **Figure 4B** shows histogram plots of CD11b^+^ Ly6G^+^ splenic neutrophils stimulated by fMLP along with unstimulated and unstained controls. **Figure 4C** shows that the number of DHR^+^ splenic neutrophils (CD11b^+^ Ly6G^+^) which were stimulated by fMLP was raised in CLP mice by approximately 50% but was not changed in splenic neutrophils taken from CLP+RvD2 mice. On the other hand, the median fluorescence intensity (MFI) of unstimulated (basal DHR^+^) and fMLP stimulated splenic neutrophils was higher in cells from RvD2 treated CLP mice (**Figure 4D**). These results suggest that RvD2 increased the efficiency of splenic neutrophils to produce ROS where the average amount of ROS produced is higher but the number of maximally stimulated cells is reduced compared to saline treated CLP mice.

### Splenic MDSCs

**Figure 5:**
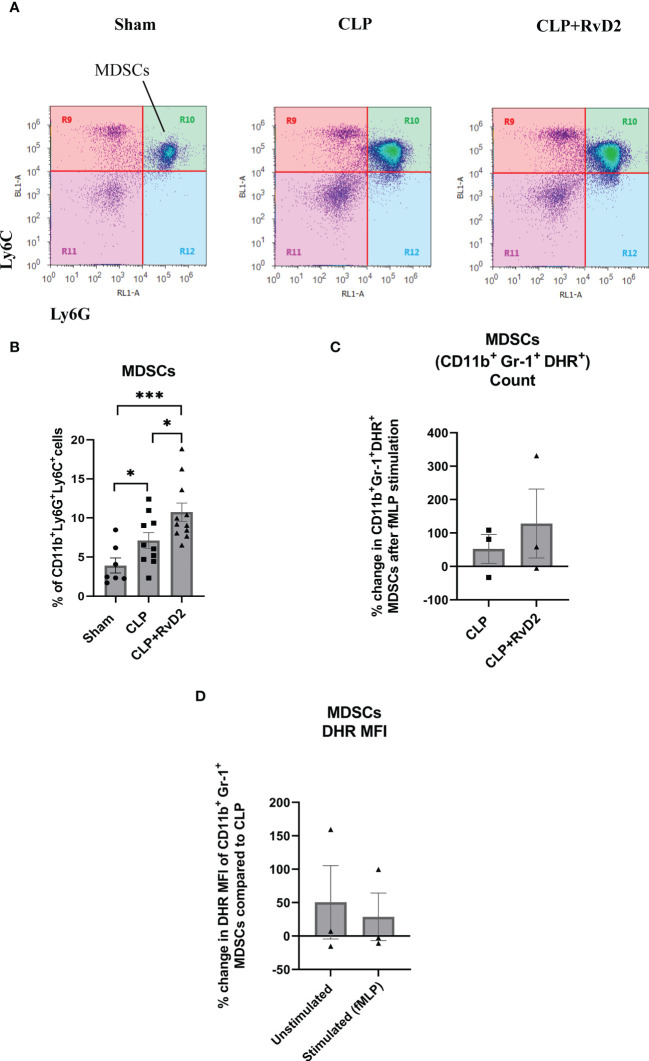
**(A)** CLP mice were given vehicle saline or RvD2 48h after surgery. Mice were sacrificed 24h later and spleen samples were taken. Myeloid-derived suppressor cells (MDSCs: CD11b^+^ Ly6C^+^ Ly6G^+^) were identified as shown. **(B)** Cells from Sham, CLP, and CLP + RvD2 groups were quantified. Splenic MDSC numbers were increased after CLP and RvD2 administration increased the numbers even further. All data are mean ± S.E.M. *P<0.05, *** P < 0.001 for n = 7-11 in all groups. **(C)** In separate experiments, the percentage change in the number of fMLP stimulated ROS producing MDSCs (CD11b^+^ Ly6G^+^ Ly6C^+^ DHR^+^) was counted for cells from CLP and CLP+RvD2 mice. RvD2 had no effect on the number of MDSC stimulated cells. **(D)** The percentage change in median fluorescence intensity (MFI) in ROS producing CD11b^+^ Ly6G^+^ Ly6C^+^ DHR^+^ cells (basal and stimulated) between CLP + RvD2 treated mice cells and cells from CLP mice was measured. RvD2 did not affect the MFI of ROS producing MDSCs.

MDSCs are a heterogenous population of immature monocytes and granulocytes which produce large quantities of ROS and NO (24, 41) which are involved in their immunosuppressive actions. They have been reported to be increased during sepsis (25–28). As production of intracellular reactive oxygen species is also involved in bacteria killing, we measured oxidative burst in the MDSCs. These cells express markers for both monocytes/macrophages (Ly6C^+^) and neutrophils (Ly6G^+^) (**Figure 5A**) (42). CLP increased the number of MDSCs in the spleen compared to sham controls. RvD2 treatment further increased the number of splenic MDSCs (**Figure 5B**). We also examined the ROS producing capacity of MDSCs in response to fMLP in cells taken from CLP mice given vehicle saline or RvD2. RvD2 did not cause any consistent change in the number of fMLP stimulated (DHR^+^) cells (**Figure 5C**). When examining alterations in MFI there was great variability and therefore there was no significant change in this parameter in basal MDSC ROS production or in fMLP stimulated ROS production (**Figure 5D**).

### Tissue and cell analyses after 2^nd^ hit with intranasal inoculation of *P. aeruginosa*

In these studies, we investigated the effects of late RvD2 administration on a 2^nd^ hit of pulmonary *P. aeruginosa*. 48h after CLP, mice were administered either RvD2 or saline vehicle. 24 after this intervention, mice were given a 2^nd^ hit where *P. aeruginosa* was administered intranasally. 24h after this 2^nd^ hit, mice were euthanized. The lungs were lavaged to obtain cells and fluid to measure bacteria load, cytokines and number of alveolar macrophages.

### Lung lavage bacteria load

**Figure 6:**
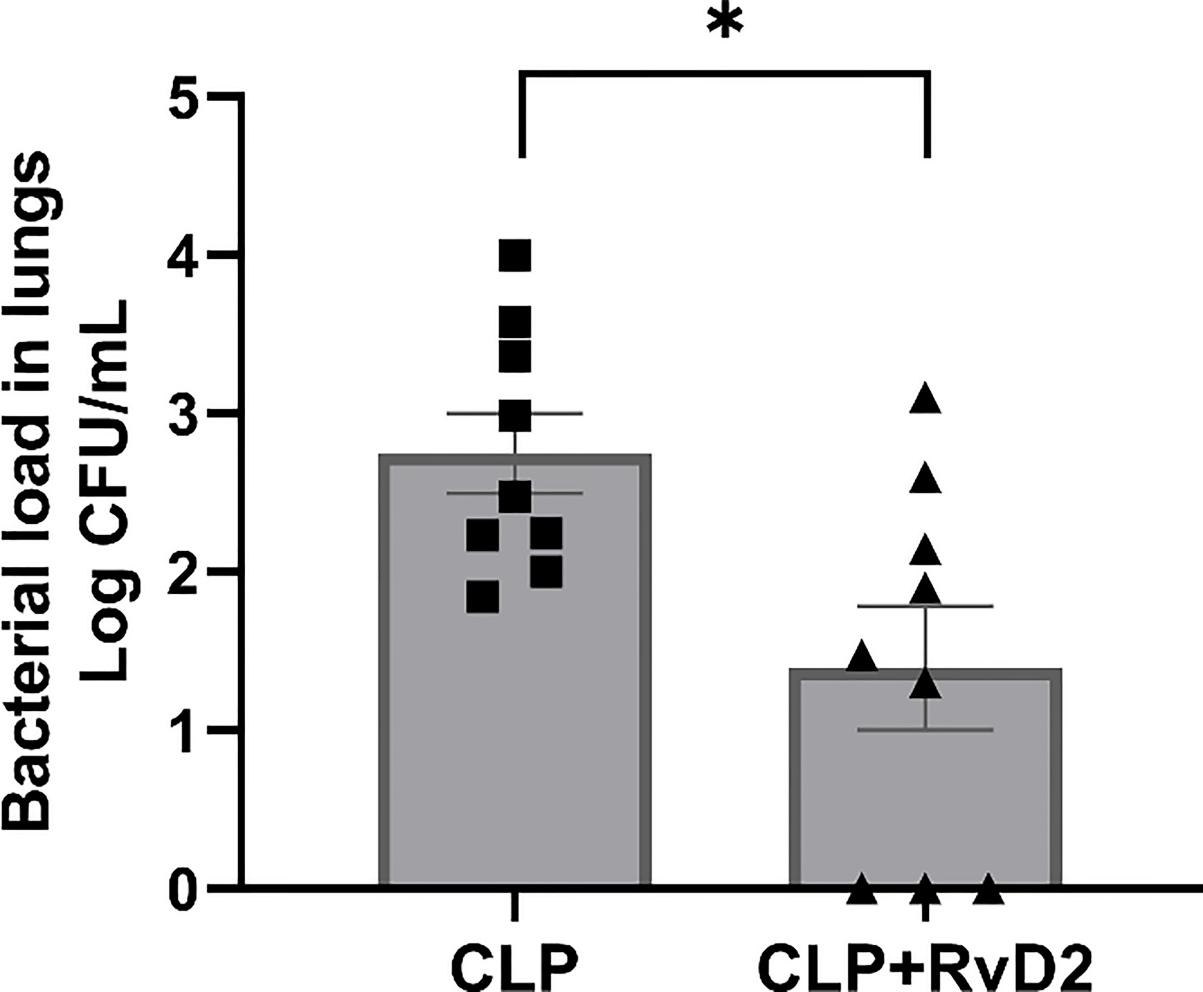
CLP surgery was performed on mice. Mice were given vehicle saline or RvD2 48h after surgery. 24h after injections, mice were given *P. aeruginosa* intranasally. 24h after the 2^nd^ hit with *P. aeruginosa*, mice were sacrificed and lungs lavaged. Lavage fluid was serially diluted and plated on TSA plates. RvD2 administration reduced lung bacteria load. Data are mean ± S.E.M. *P<0.05 for n = 9 in all groups.

RvD2 treatment 24h before the second hit of pulmonary *P. aeruginosa* significantly reduced lung bacteria load compared to vehicle treated mice (**Figure 6**).

### Lung lavage cytokines

**Figure 7:**
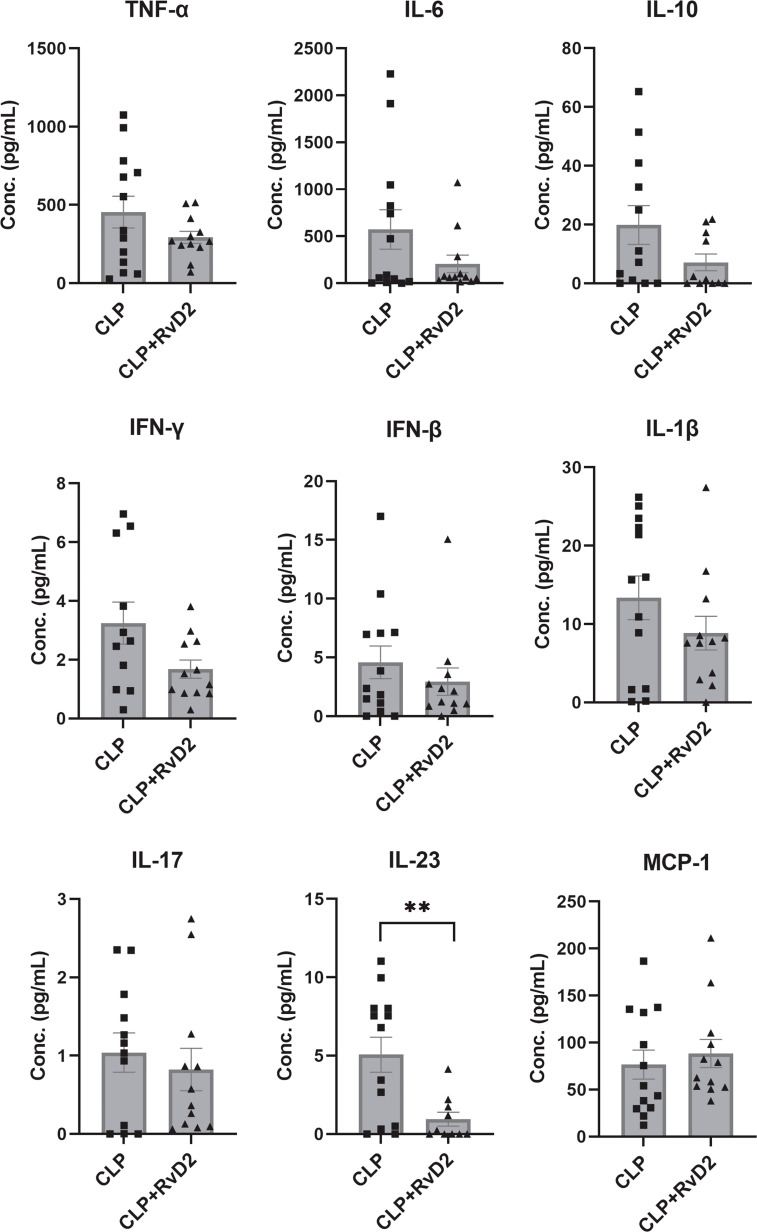
CLP surgery was performed on mice. Mice were given vehicle saline or RvD2 48h after surgery. 24h after injections, mice were given *P. aeruginosa* intranasally. 24h after the 2^nd^ hit of p. aeruginosa, mice were sacrificed and lungs lavaged. Using flow cytometry, cytokines in lungs including TNF-α, IL-6, IL-10, IFN-γ, IFN-β, IL-1β, IL-17, IL-23, and MCP-1 were quantified by bead-based immunoassay. RvD2 significantly reduced lung lavage fluid IL-23 levels. Data are mean ± S.E.M. ** P < 0.01 for n = 10 – 12 in all groups.

We measured lung lavage fluid cytokine levels as an index of lung inflammatory response. Lavage fluid cytokine levels were measured by bead-based immunoassay (Legendplex Flow cytometry). Lung lavage cytokine levels were similar in the 2 groups of mice with the exception of IL-23 (**Figure 7**). RvD2 treatment significantly reduced lung lavage levels of IL-23.

### Alveolar macrophages

**Figure 8:**
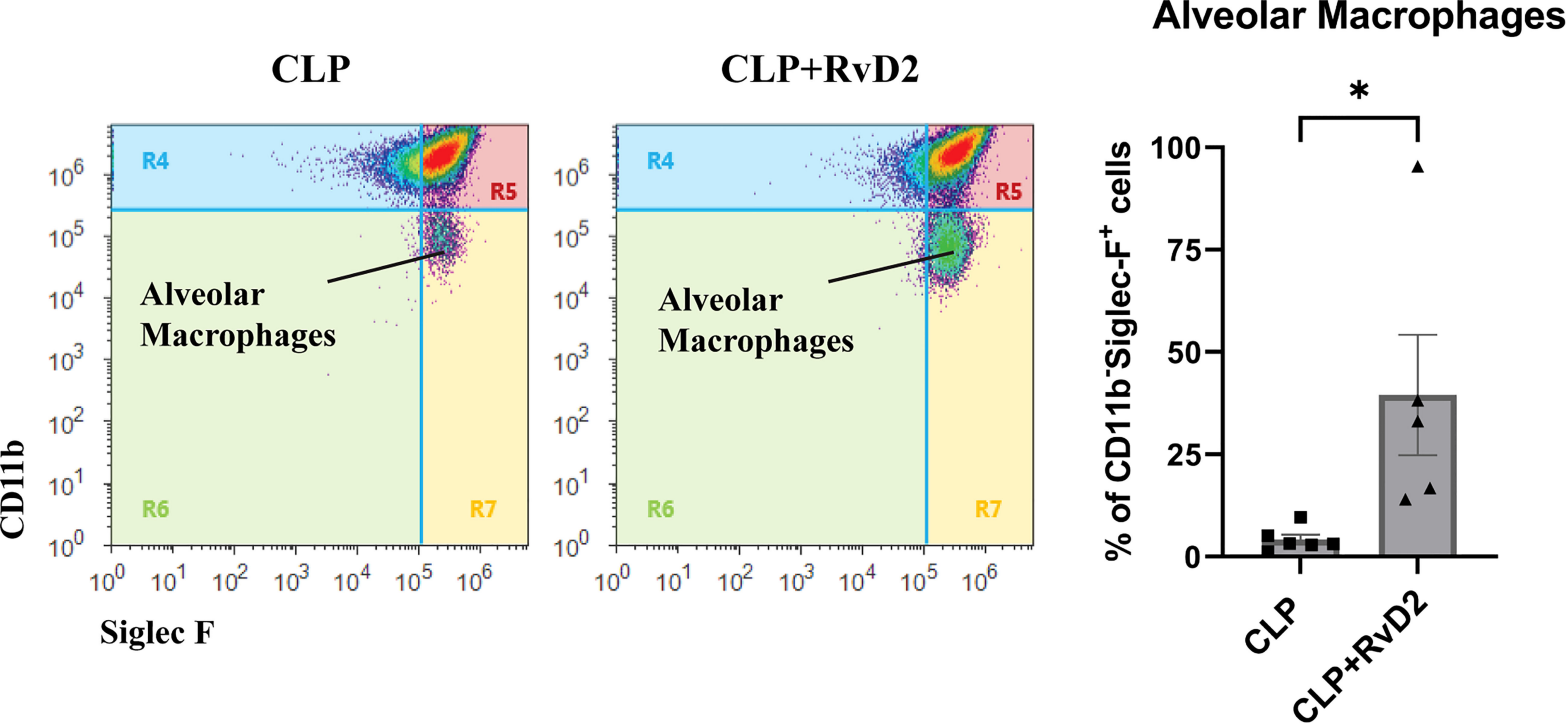
CLP surgery was performed on mice. Mice were given vehicle saline or RvD2 48h after surgery. 24h after injections, mice were given *P. aeruginosa* intranasally. 24h after the 2^nd^ hit of *P. aeruginosa*, mice were sacrificed and lungs were lavaged. Using flow cytometry, non-inflammatory alveolar macrophages (CD11b^-^ Siglec F^+^) were counted in lung lavage. There was an increase in non-inflammatory alveolar macrophages in lavage fluid of RvD2 treated mice. Data are mean ± S.E.M. * P < 0.05 for n = 5 for both groups.

Non-inflammatory alveolar macrophages (CD11b^-^SiglecF^+^) are resident macrophages which do not express CD11b, a membrane protein involved in leukocyte migration and inflammation (34). These cells can therefore be considered as indicators of infection/inflammation resolution in the lungs.Lung lavage contained approximately 3.38 ± 1.21 X 10^6^ cells (CLP) and 1.23 ± 0.55 X 10^6^ cells (CLP + RvD2) respectively. We measured the numbers of non-inflammatory alveolar macrophages (CD11b^-^ SiglecF^+^) in lavage fluid after RvD2 or saline vehicle administration as an index of infection resolution. RvD2 administration increased the number of non-inflammatory alveolar macrophages in lung lavage fluid (**Figure 8**).

## Discussion

This study provides evidence of RvD2 mediated cellular and molecular mechanisms underlying bacterial clearance in blood and lungs in a 2-hit infection model of peritonitis, followed by secondary lung infection. Peritonitis in our model was triggered by leakage of fecal bacteria from the punctured cecum into the sterile peritoneum during the first 48 hours, causing an increase in inflammatory cytokines in the CLP group compared to sham mice. When RvD2 was administered during this late time point, we observed an increase in splenic neutrophil and MDSCs. Prior RvD2 treatment, also increased free radical generation (DHR^+^ MFI) in CD11b^+^Ly6G^+^ splenic neutrophils upon fMLP stimulation. However, free radical production in MDSCs was not altered. The late administration of RvD2 reduced *P. aeruginosa* lung load and decreased lavage IL-23 levels. RvD2 increased non-inflammatory alveolar macrophage numbers. These latter results suggest that not only does RvD2 play an important role in resolution of acute infection/inflammation but it actively prevents chronic inflammation directly by reducing IL-23.

We have previously reported that RvD2 reduced blood bacteria load in the 2-hit model of CLP and secondary lung infection without altering plasma IL-6, IL-10 and MIP-2 levels (17). In the current study we extend our measurements to include cytokines of the adaptive immune system such as IFN-γ, IL-17, IFN-β and IL-12. At this time point, plasma levels of IFN-β which suppresses pro-inflammatory cytokine release (43), was higher in CLP mice than sham controls while IL-12 one of whose functions is to help differentiate CD4 T-cells into TH1 cells (44), was not significantly increased. Similarly, IL-17 a pro-inflammatory cytokine involved in chronic autoimmune disorders (45) was not increased. RvD2 did not affect the levels of any cytokines. Taken together with our preliminary data which shows that in this model, liver IL-6 is not raised while the anti-inflammatory cytokine IL-10 is significantly elevated, the results suggest that at this late time point after CLP, the overall state of the adaptive immune system was in post pro-inflammatory phase. RvD2 did not affect this state.

In general, SPMs reduce neutrophil transmigration, promote neutrophil apoptosis but increase phagocytic ability (46–51). This attenuation of neutrophil migration is an important part of the inflammation resolution process to aid the host in restoring homeostasis and avoiding chronic inflammation. With the reduction in neutrophil migration to the site of injury, it is thought that a major mechanism by which neutrophils can aid in bacterial clearance is through an increase in phagocytic ability. Indeed, we have previously shown that Lipoxin A4 (LxA4) can increase blood neutrophil phagocytic ability and RvD2 can increase lung alveolar monocyte/macrophage phagocytic ability (17, 52, 53).

The spleen plays a major role in bacterial clearance and immune regulation where splenic macrophages aid in bacterial clearance by phagocytosis and together with dendritic cells present antigen to initiate T-cell activation, differentiation and expansion as part of the adaptive immune response (54, 55). In addition, splenic B-cells can also act as antigen cells for the production of antibodies as another part of the adaptive immune response. There has however been very little work done on splenic neutrophils. In their seminal publication, Kubes and co-authors (29) reported that mature and immature neutrophils were important for bacterial clearance in the spleen. In addition, *in vitro* studies have shown that RvD2 can restore the directionality of neutrophils after burns (56). Our results showing both an increase in number and an increase in free radical production coupled with a significant decrease in blood bacteria load, is consistent with the notion that splenic neutrophil accumulation improved blood bacteria clearance. The reduction in blood bacteria load together with any significant increase in plasma cytokines indicated RvD2 may directly affect cell function without changing inflammatory signaling. Taken together, our results suggest that RvD2 given as late as 48h after CLP-induced infection increased blood bacteria clearance at least partly through an increase in splenic neutrophil number and activity.

The role of MDSCs in infectious disease is unclear. This heterogenous population of cells has been shown to be increased in various infectious disease models such as CLP-induced sepsis (26, 27), *Porphyromonas gingivalis* infection (57), and *Trypanosoma cruzi* infection (58). In these disease models the MDSCs have been shown to suppress T-cell proliferation (25), inhibit ConA-induced splenocyte proliferation and suppress adaptive immunity through the Programmed Cell Death 1 receptor (PD1)/Programmed Cell Death Receptor 1 Ligand (PDL1) axis (23). MDSCs obtained from early (3 days) CLP-sepsis appeared to express pro-inflammatory cytokines and nitric oxide while those isolated late (day 12) in sepsis expressed anti-inflammatory cytokines (IL-10) and TGFβ. Interestingly adoptive transfer of late MDSCs into CLP mice early in disease infection development, decreased overall mortality and vice versa (27). In the *Trypanosoma cruzi* infection model *in vivo* depletion of MDSCs actually led to increased pro-inflammatory cytokine production and mortality (58). These latter reports highlight the heterogeneity and activity of these cells during infection. In this study, we show that there is an increase in MDSCs in the spleen of RvD2 treated mice. These cells do not appear to be significantly pro-inflammatory as there was no significant increase in plasma cytokine levels. The free radical production from these cells was not increased and circulating plasma cytokine levels were not altered but blood bacteria load was significantly reduced. These results suggest a mechanism by which RvD2 promotes host defense, is to increase bacterial clearance by increasing splenic MDSC number.

In previous studies, we have shown that RvD2 reduced bacterial load and lung inflammation after a 2^nd^ hit with intranasal *P. aeruginosa* in this model (17). In the current studies, we wished to examine tissue immunological changes in the lung after intranasal *P. aeruginosa* administration. To do this we measured bronchoalveolar lavage fluid levels of cytokines. There was a strong trend towards a lower cytokine level in RvD2 treated mice, but only IL-23 levels were significantly lower. IL-23 is a member of the IL-12 family. The cytokine is mainly secreted by activated macrophages peripheral tissues such as lungs, skin and intestines (59). It is involved in T-cell differentiation to the Th-17 phenotype (60) and has been implicated in the inflammatory processes of several autoimmune disorders such as psoriasis, inflammatory bowel disease and arthritis (61–63). On the other hand, IL-23 deficient mice were unable to clear *Klebsiella pneumoniae* lung infections (64). These reports suggest that certain levels of IL-23 are essential for bacterial clearance while sustained high amounts lead to chronic inflammatory disorders. Our results which show a decrease of IL-23 with RvD2 administration suggest that RvD2 lowers the possibility of chronic infection through an IL-23-dependent mechanism within the adaptive immune system. This finding is consistent with previous work showing that RvD1 and RvD2 are important modulators of T-cell responses (65). The mechanism of increased IL-23 secretion has been reported to be through the coordinated actions of TGFβ with IL-6 (66) and is a subject of ongoing investigation in our laboratory.

Bacterial clearance in the lung during infection is dependent on alveolar macrophage activity and depletion of alveolar macrophages reduces lung bacterial clearance (67, 68). We and others have shown that resolvins enhance lung bacterial clearance by increasing macrophage activity (17, 69, 70). In this study we show that non-inflammatory alveolar macrophage numbers are low 24h after *P. aeruginosa* administration. RvD2 significantly increased their numbers. Alveolar macrophage numbers have been reported to have been decreased after influenza (68) or *P. carinii* infections (71). This decrease was associated with a decrease in subsequent pathogen clearance. The decrease in alveolar macrophages may in part be due to macrophage apoptosis which is a process which helps in bacterial killing (72) but excessive macrophage amounts of apoptosis may leave the host unable to clear bacteria efficiently. Our data clearly showing that lung bacteria load was reduced and there was a decrease in a pro-inflammatory cytokine IL-23 in the lavage fluid together with stration, provide evidence of more efficient infection resolution with better bacterial clearance and inhibition of chronic inflammatory response.

In summary, using a clinically relevant model of primary peritoneal infection followed by secondary lung infection, our studies have uncovered host defense mechanisms by which RvD2 promotes bacterial clearance and inhibits potential chronic inflammation. These findings provide support for further mechanistic studies into the use of RvD2 in primary and secondary infections.

## Data availability statement

The raw data supporting the conclusions of this article will be made available by the authors, without undue reservation.

## Ethics statement

The animal study was reviewed and approved by IACUC of Rowan University.

## Author contributions

PKS performed all flow cytometric studies and prepared *P. aeruginosa* for intranasal administration. All surgery was performed by KY with assistance from JW. All injections and bacteria load plating was performed by JW. RvD2 was prepared by AR and BS. The studies were designed by KY and PKS. The paper was written by KY and PKS. All authors contributed to the article and approved the submitted version.

## Funding

The work was supported by NIAID (RO1AI128202) to KY.

## Acknowledgments

We thank our lab members Julianne Thornton, and Rachael Wilson for helping us with monitoring mice.

## Conflict of interest

The authors declare that the research was conducted in the absence of any commercial or financial relationships that could be construed as a potential conflict of interest.

## Publisher’s note

All claims expressed in this article are solely those of the authors and do not necessarily represent those of their affiliated organizations, or those of the publisher, the editors and the reviewers. Any product that may be evaluated in this article, or claim that may be made by its manufacturer, is not guaranteed or endorsed by the publisher.

## Supplementary material

Supplementary Figure 1 Gating Strategy: Myeloid-derived suppressor cells were gated based on size (FSC) and granularity (SSC), singlets, live cells, expression of CD11b, Ly6G, and Ly6C in a quadrant gate. Similarly alveolar macrophages were gated based on size (FSC) and granularity (SSC), singlets, live cells, expression of SiglecF and non-expression of CD11b in a quadrant gate.

Supplementary Figure 2 Liver Tissue cytokine levels. Sham or CLP surgery was performed on mice. CLP mice were given vehicle saline or RvD2 48h after surgery. Mice were sacrificed 24h later and liver samples were taken and homogenized in 2mL of T-PER buffer containing protease inhibitors and homogenized according to our protocol (17). Cytokines of liver extracts were measured by ELISA (Thermofisher Scientific). Liver IL-6 levels were not changed between the 3 groups. Macrophage inflammatory protein-2 (MIP2) levels were increased in livers of CLP and CLP + RvD2 mice compared to sham controls. Similarly, Liver Il-10 levels were increased in CLP and CLP + RvD2 mice compared to sham controls. * P < 0.05 for n = 3-7 mice in all groups.
