# Supplementary material for: Resolvin D2 induces anti-microbial mechanisms in a model of infectious peritonitis and secondary lung infection
Source: Front Immunol. 2022 Dec 1;13:1011944. doi: 10.3389/fimmu.2022.1011944 (PMC9754689; doi:10.3389/fimmu.2022.1011944)

Supplement Figure 1: GATING STRATEGY

MYELOID-DERIVED SUPPRESSOR CELLS (MDSCs): CD11b⁺ Ly6C⁺ Ly6G⁺

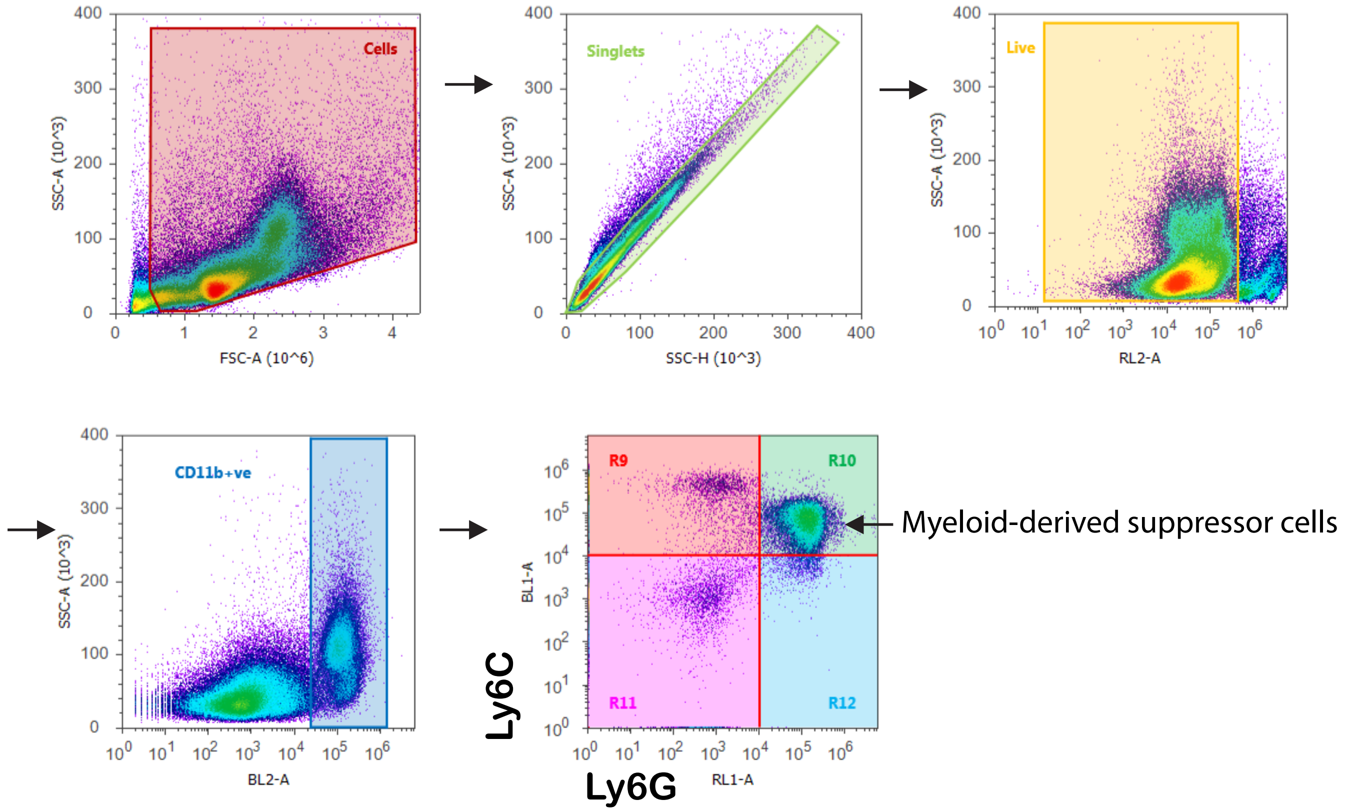

ALVEOLAR MACROPHAGES (AM) : CD11b⁻ SiglecF⁺

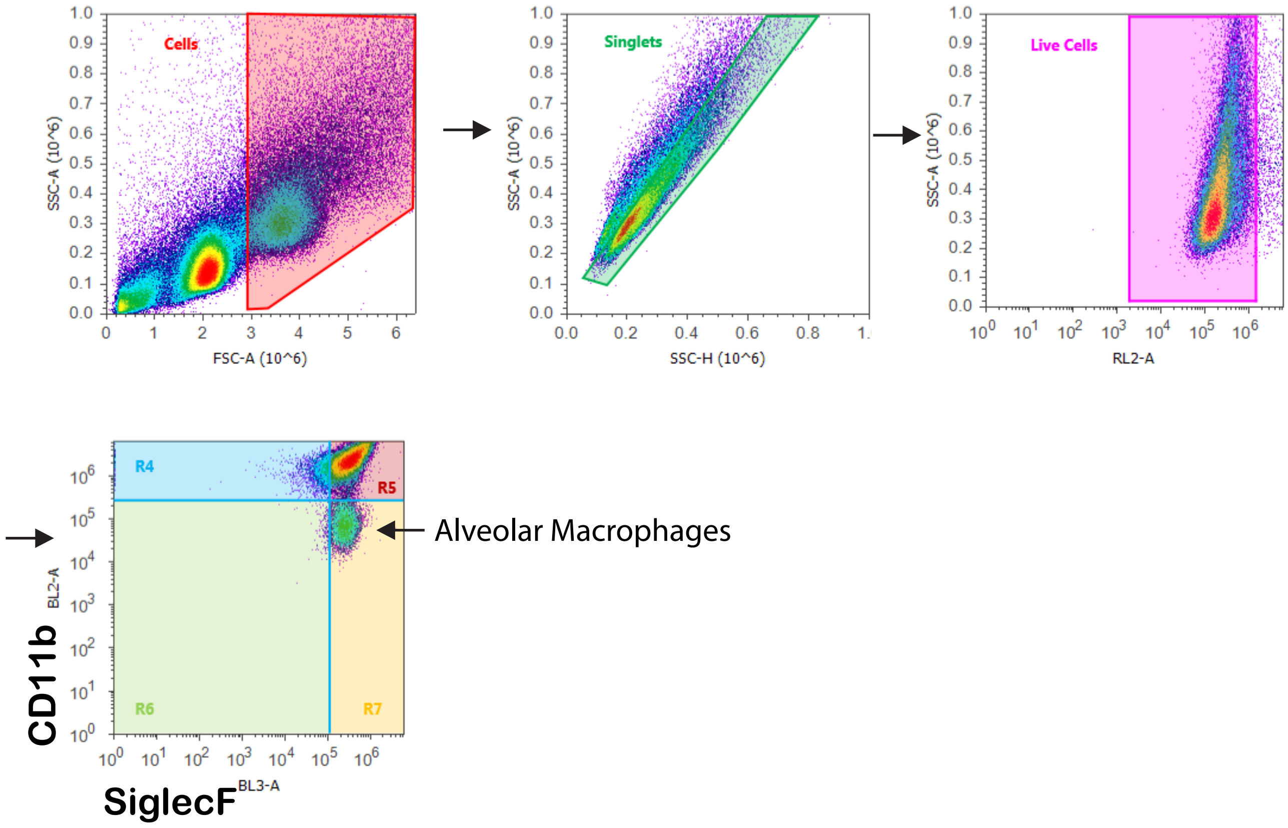

Supplement: Supplementary Figure 1 — Gating Strategy: Myeloid-derived suppressor cells were gated based on size (FSC) and granularity (SSC), singlets, live cells, expression of CD11b, Ly6G, and Ly6C in a quadrant gate. Similarly alveolar macrophages were gated based on size (FSC) and granularity (SSC), singlets, live cells, expression of SiglecF and non-expression of CD11b in a quadrant gate. [file Image_1.pdf]
