# Supplementary figures and images for: Resolvin D2 induces anti-microbial mechanisms in a model of infectious peritonitis and secondary lung infection
Source: Front Immunol. 2022 Dec 1;13:1011944. doi: 10.3389/fimmu.2022.1011944 (PMC9754689; doi:10.3389/fimmu.2022.1011944)

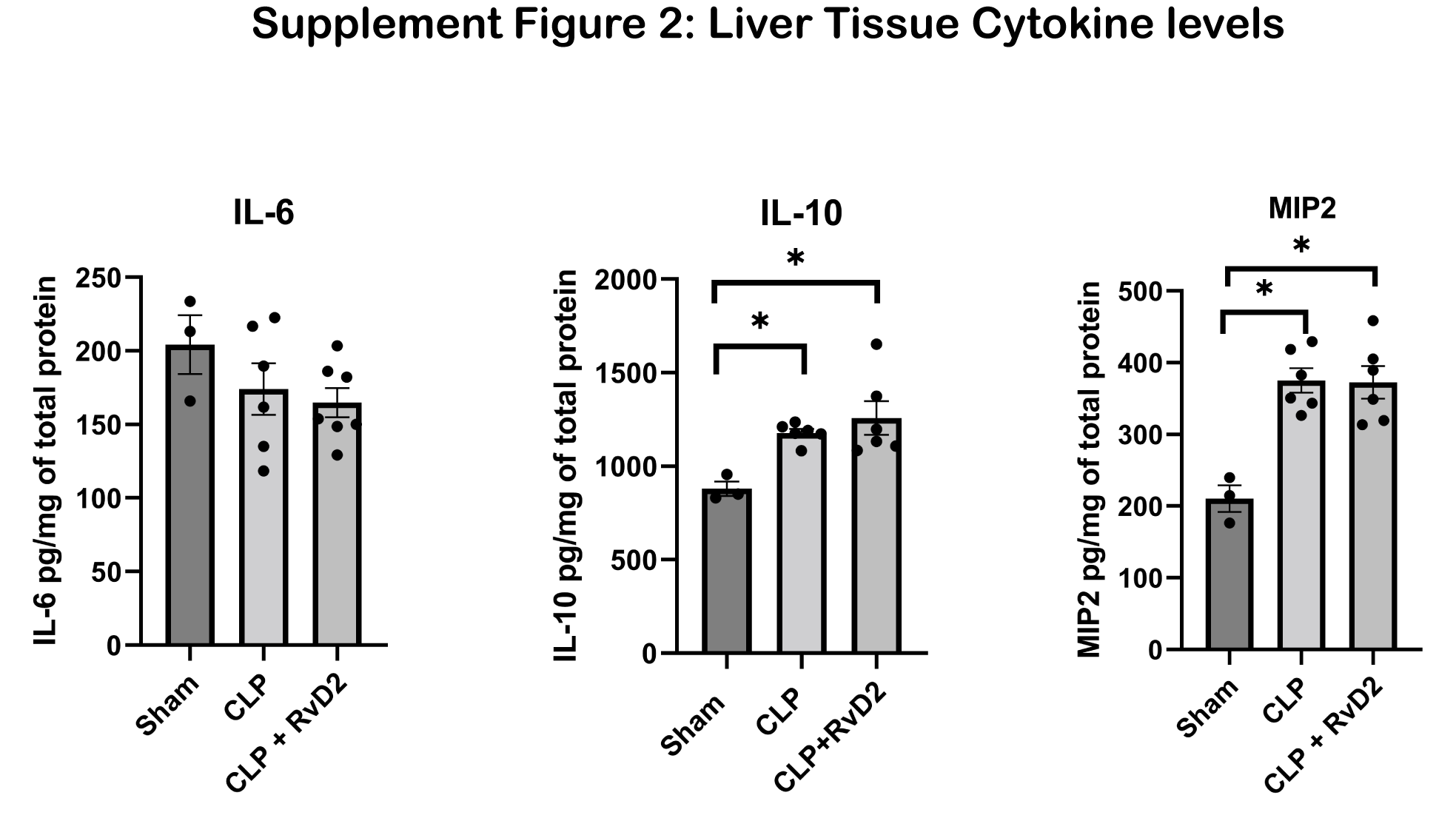

Supplement: Supplementary Figure 2 — Liver Tissue cytokine levels. Sham or CLP surgery was performed on mice. CLP mice were given vehicle saline or RvD2 48h after surgery. Mice were sacrificed 24h later and liver samples were taken and homogenized in 2mL of T-PER buffer containing protease inhibitors and homogenized according to our protocol (17). Cytokines of liver extracts were measured by ELISA (Thermofisher Scientific). Liver IL-6 levels were not changed between the 3 groups. Macrophage inflammatory protein-2 (MIP2) levels were increased in livers of CLP and CLP + RvD2 mice compared to sham controls. Similarly, Liver Il-10 levels were increased in CLP and CLP + RvD2 mice compared to sham controls. * P < 0.05 for n = 3-7 mice in all groups. [file Image_2.tif]
